# Emergence of *Salmonella enterica* carrying *bla*_OXA-181_ carbapenemase gene, Italy, 2021 to 2024

**DOI:** 10.2807/1560-7917.ES.2025.30.13.2500175

**Published:** 2025-04-03

**Authors:** Luca Bolzoni, Erika Scaltriti, Chiara Bracchi, Sara Angelone, Ilaria Menozzi, Roberta Taddei, Patricia Alba, Virginia Carfora, Elena Lavinia Diaconu, Marina Morganti, Alessandra Dodi, Melissa Berni, Laura Manni, Massimiliano Vinci, Martina Tambassi, Laura Mazzera, Irene Venturelli, Simone Ambretti, Antonio Battisti, Stefano Pongolini

**Affiliations:** 1 Risk Analysis and Genomic Epidemiology Unit, Istituto Zooprofilattico Sperimentale della Lombardia e dell'Emilia-Romagna, Parma, Italy; 2 Bologna Unit, Istituto Zooprofilattico Sperimentale della Lombardia e dell'Emilia-Romagna, Bologna, Italy; 3 Department of General Diagnostics, National Reference Laboratory for Antimicrobial Resistance, Istituto Zooprofilattico Sperimentale del Lazio e Della Toscana “M. Aleandri”, Rome, Italy; 4 Clinical Microbiology, Azienda Ospedaliera-Universitaria - Policlinico di Modena, Modena, Italy; 5 Microbiology Unit, IRCCS Azienda Ospedaliero-Universitaria di Bologna, Bologna, Italy; 6 Department of Medical and Surgical Sciences, Section of Microbiology, University of Bologna, Bologna, Italy

**Keywords:** *Salmonella enterica*, *bla_OXA-181_*, carbapenemase, antimicrobial resistance

## Abstract

Between 2021 and 2024, we detected carbapenemase gene *bla_OXA-181_* in 16 of 11,398 *Salmonella enterica* (SE) isolates: 10 SE 1,4,[5],12:i:-, three Bovismorbificans, two London and one Rissen from pigs, humans, pork meat and wild roe deer. The gene was first detected in pig isolates, later in humans, suggesting zoonotic transmission. Phylogenetic analysis indicated that horizontal transfer, mainly through plasmids, contributed to the spread. These findings highlight a possible emerging public health threat and the importance of One Health surveillance.

In 2021, carbapenemase-coding *bla*_OXA-181_ gene [1] was detected in isolates of indicator *Escherichia coli* in pigs in northern Italy [2]. To date, *bla*_OXA-181_ has not been detected in *Salmonella enterica* (SE) isolates in the European Union (EU) [3]. We aimed to investigate the dissemination of SE carrying *bla*_OXA-181_ in isolates from humans, animals and food in northern Italy.

## Surveillance system and data analysis

In northern Italy, the Istituto Zooprofilattico Sperimentale della Lombardia e dell’Emilia-Romagna (IZSLER) is the official laboratory for genomic surveillance of salmonellosis in humans in the Administrative Region of Emilia-Romagna (4.5 million residents). In addition, IZSLER characterises *Salmonella* isolates from animal and food samples as part of official control and private testing in Emilia-Romagna and the neighbouring Region of Lombardy. In total, 62% of the Italian pig and 38% of the cattle population reside in these two regions [4].

Between January 2021 and December 2024, we whole genome sequenced (WGS) all submitted *Salmonella* isolates from humans (n = 2,824; 2021: n = 669; 2022: n = 651; 2023: n = 723; 2024: n = 781) and animals and food (n = 8,574; 2021: n = 995; 2022: n = 1,960; 2023: n = 2,751; 2024: n = 2,868) from these two regions for identification of *Salmonella* clusters and tracking sources of infection. The animal samples were mostly from livestock but also from wildlife and pets.

In 2024, we started screening genomes for antimicrobial resistance (AMR) genes, beginning with isolates from January 2021. We used Resfinder [5,6] for identification of resistance genes and detected *bla*_OXA-181_ gene in 16 (0.14%) isolates, four from 2023 and 12 from 2024. Using the R statistical programme version 3.5.2 (https://www.r-project.org/), we tested the significance of the increase using logistic regression, and it was statistically significant (p = 0.00218).

**Figure 1:**
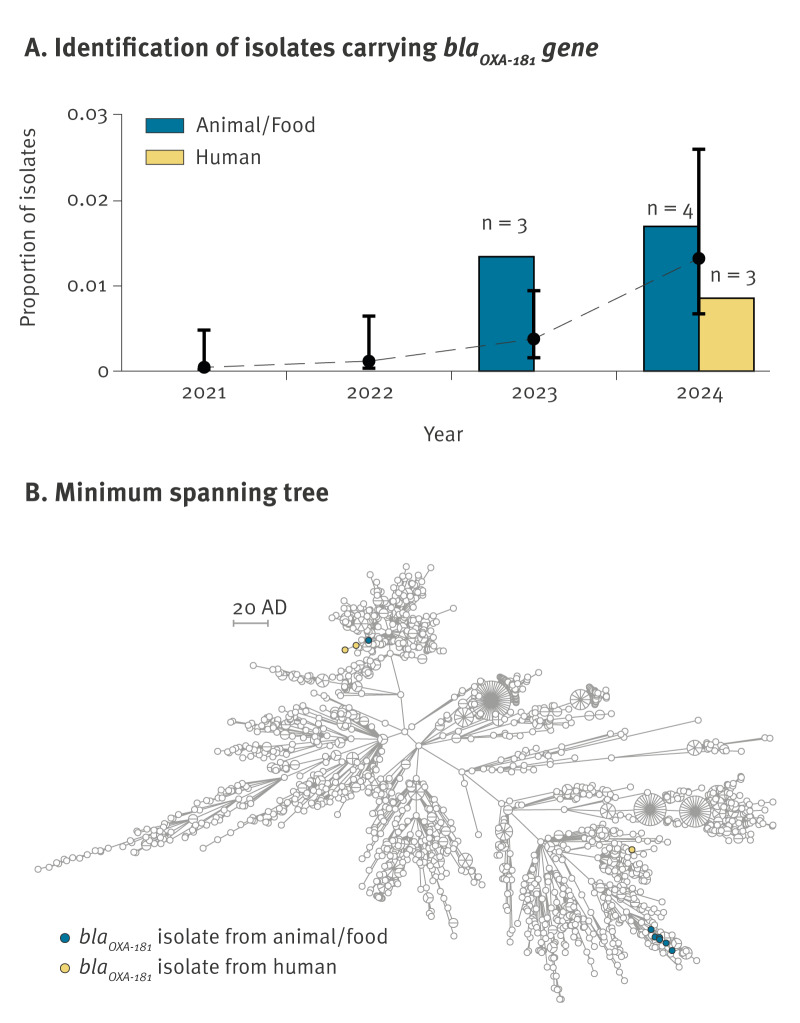
Identification of *Salmonella enterica* 1,4,[5],12:i:- isolates with *bla*_OXA-181_ gene from humans, animals and food, northern Italy, 2021–2024 (n = 1,918)

The isolates were from monophasic variant of *S*. Typhimurium (SE 1,4,[5],12:i:- (MVST)) (n = 10), *S*. Bovismorbificans (n = 3), *S*. London (n = 2) and *S*. Rissen (n = 1) (Table). Twelve of these isolates were from pigs or pork, three from humans and one isolate was from a wild roe deer. The *bla_OXA-181_*-positive MVST isolates showed a statistically significant increase (p = 0.0011) (Figure 1).

**Table:** Characterisation of *Salmonella enterica* isolates from humans, animals and food carrying the *bla_OXA-181_* gene, northern Italy, 2023–2024 (n = 16)

Characteristics						MIC values (mg/L)^a^			
Isolate ID	Isolation date	Source	Serovar	ST	*bla_OXA-181_* localisation	MERO	ETP	IMI	TRM
2023–050284–001–01	Feb 2023	Pig	MVST	34	Plasmid IncX1	1	2	0.5	> 128
2023–257642–001–01	Aug 2023	Wild roe deer	SR	469	Chromosome	0.25	0.25	0.5	> 128
2023–307061–002–01	Oct 2023	Pig^b^	MVST	34	Plasmid IncX1	0.5	1	0.5	> 128
2023–403546–001–01	Dec 2023	Pig^b^	MVST	34	Plasmid IncX1	0.5	1	0.5	> 128
2024–005381–001–01	Jan 2024	Pig^b^	MVST	34	Plasmid IncX1	0.5	2	0.5	> 128
2024–005381–003–01	Jan 2024	Pig^b^	MVST	34	Plasmid IncX1	0.5	2	0.5	> 128
2024–005381–004–01	Jan 2024	Pig^b^	MVST	34	Plasmid IncX1	0.5	2	0.5	> 128
2024–132632–002–01	Apr 2024	Pork	SB	2640	Plasmid IncX3	0.25	0.5	1	> 128
2024–132632–004–01^c^	Apr 2024	Pork	SB	2640	Plasmid IncX3	0.25	0.5	1	> 128
2024–132632–005–01^c^	Apr 2024	Pork	SB	2640	Plasmid IncX3	0.25	0.5	1	> 128
2024–124985–001–01	Apr 2024	Human	MVST	34	Plasmid IncX1	1	2	0.5	> 128
2024–142809–005–01	May 2024	Human	MVST	34	Plasmid IncX1	1	2	0.5	> 128
2024–074445–001–01	Mar 2024	Human	MVST	34	Plasmid IncX3	0.25	1	0.5	> 128
2024–271534–004–01^c^	Sep 2024	Pig	SL	155	Plasmid IncX1	0.25	0.5	0.25	> 128
2024–271534–005–01	Sep 2024	Pig	SL	155	Plasmid IncX1	0.25	0.5	0.25	> 128
2024–326624–003–01	Oct 2024	Pig^b^	MVST	34	Plasmid IncX1	0.5	1	0.5	> 128

ECOFF: epidemiological cutoff; ETP: ertapenem; ID: identification code; IMI: imipenem; MERO: meropenem; MIC: minimum inhibitory concentration; MVST: monophasic variant of *Salmonella* Typhimurium, SB: *Salmonella* Bovismorbificans, SL: *Salmonella* London; SR: *Salmonella* Rissen; ST: sequence type; TRM: temocillin.

^a^ The following ECOFFs were used to determine resistance: meropenem (resistant (R) > 0.125 mg/L); ertapenem (R > 0.064 mg/L); imipenem (R > 1) and temocillin (R > 16 mg/L).

^b^ Isolates were obtained from samples from different farms belonging to the same agricultural company.

^c^ Isolates sequenced with Illumina short-read only.

Panel A. The black line and the dots represent the estimated proportion of *Salmonella enterica* 1,4,[5],12:i:- isolates carrying *bla_OXA-181_* as a function of time, through logistic regression. The proportion of *bla_OXA-181_*-carrying isolates showed a statistically significant increase (p = 0.0011). A total of 1,157 isolates from humans were analysed: 2021: n = 280; 2022: n = 256; 2023: n = 270 and 2023: n = 351. A total of 761 isolates from animals and food were analysed: 2021: 89; 2022: n = 214; 2023: n = 223 and 2024: n = 235).

Panel B. Minimum spanning tree was based on the core genome multilocus sequence typing (MLST) (Innuendo scheme) of all *Salmonella enterica* 1,4,[5],12:i:- of the study. The bar corresponds to a distance of 20 alleles (AD).

We determined minimum inhibitory concentrations (MIC) by broth microdilution using EUVSEC2 96-well microtitre plates (Trek Diagnostic Systems, Westlake, the United States). The results were interpreted according to epidemiological cutoffs (ECOFFs) [7,8] or clinical breakpoints (CB) of the EUCAST [9]. The following ECOFFs and CBs were used: meropenem (ECOFF: resistant (R) > 0.125; CB: R > 8); ertapenem (ECOFF: R > 0.064 mg/L; CB: R > 0.5 mg/L); imipenem (ECOFF: R > 1 mg/L; CB: R > 4 mg/L) and temocillin (TRM; ECOFF: R > 16 mg/L). All isolates were resistant to meropenem, ertapenem and temocillin (ECOFF), while 10 isolates were also clinically resistant to ertapenem (Table).

## Characterisation of isolates with *bla*_OXA-181_

**Figure 2:**
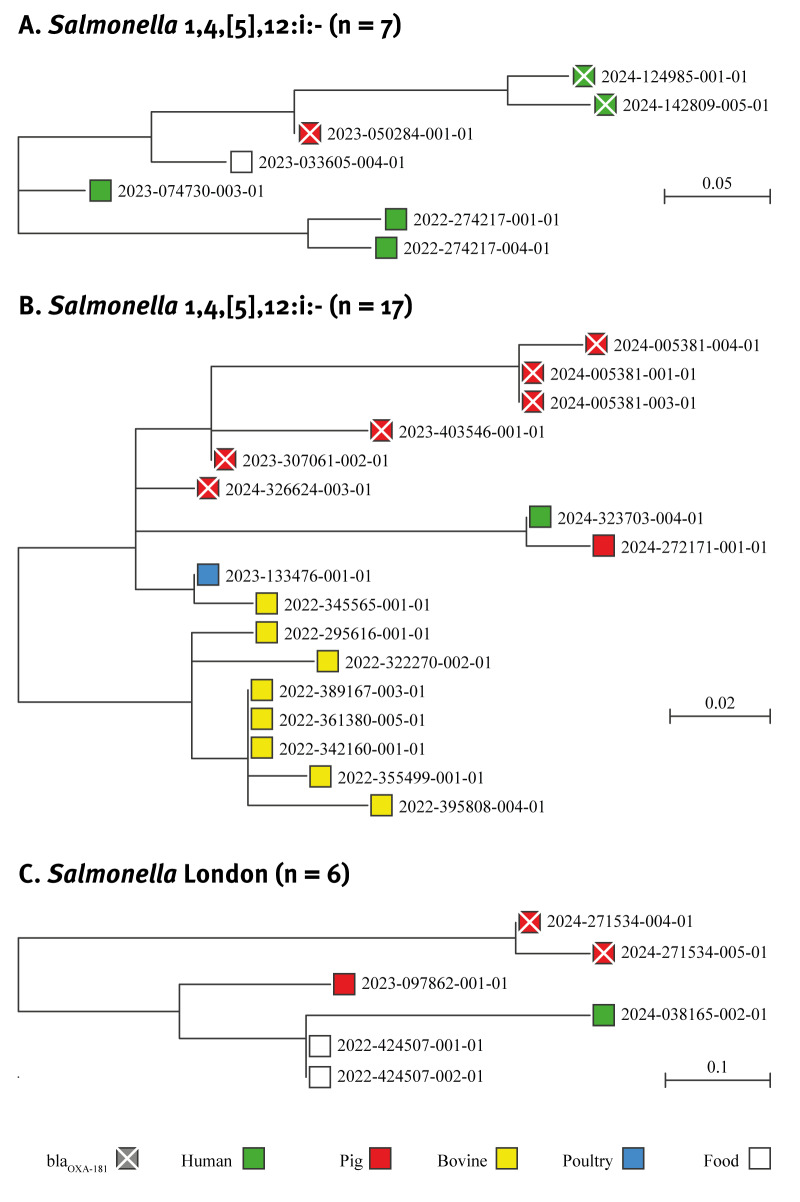
Maximum likelihood phylogenetic trees of clones of *Salmonella enterica* including isolates carrying *bla*_OXA-181,_ northern Italy, 2022–2024 (n = 30)

**Figure 3:**
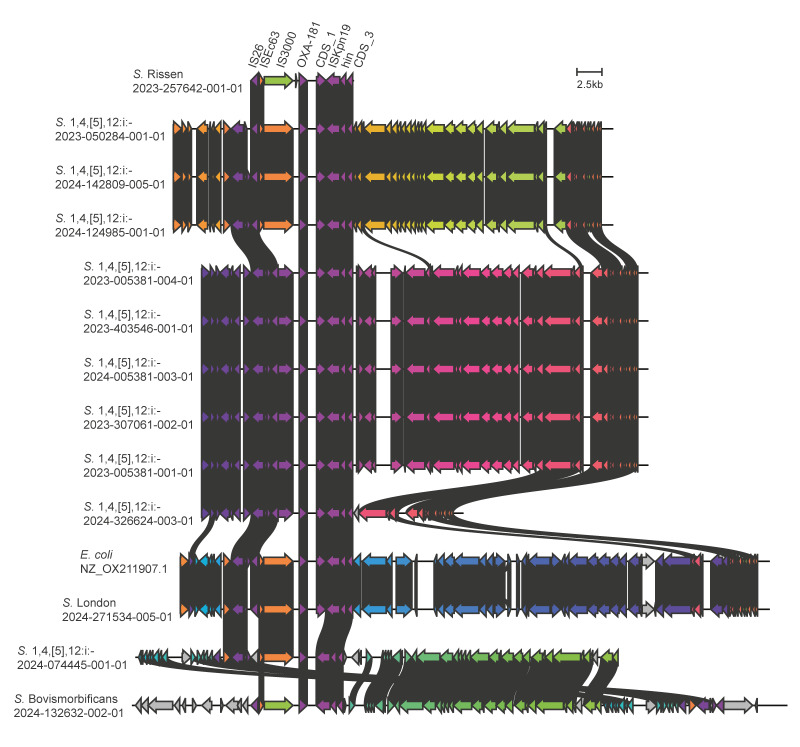
Description of plasmids carrying *bla*_OXA-181_ gene in 12 *Salmonella enterica* isolates and one *Escherichia coli* isolate, northern Italy, 2023–2024

We generated closed assemblies of the genomes by combining Illumina short-reads and Oxford Nanopore long-reads [10]. We analysed the phylogeny of the *bla*_OXA-181_-carrying isolates including all 11,398 genomes surveyed from 2021 to 2024 in the analysis. Overall, the isolates belonged to six clearly distinct and distant lineages [11], and the *bla*_OXA-181_-carrying isolates of MVST belonged to three different clones (Figure 1B). In several cases, the isolates carrying *bla*_OXA-181_ were part of SE clones, endemic in northern Italy, in which also isolates without the gene were present, and these were generally older than the ones with the AMR gene. These findings strengthen the hypothesis of horizontal acquisition of *bla*_OXA-181_ along the evolutionary pathway. Three *bla*_OXA-181_-carrying isolates of MVST, two from humans and one from pig, belonged to the same clone together with other food and human isolates (2–13 SNPs), all devoid of *bla*_OXA-181_ (Figure 2A). Consistently, these three *bla*_OXA-181_-positive isolates had the same IncX1 plasmid harbouring *bla*_OXA-181_, i.e. with 100% identity and similarity (Figure 3)_._

Colours represent the compartments of the origin of the isolates.

The figure was generated with Clinker.

The alignment also shows the chromosomal locus including *bla*_OXA-181_ of *S*. Rissen 2023–257642–001–01 from this study and plasmid pMOL6975 (The National Center for Biotechnology Information (NCBI) reference NZ_OX211907.1) from *E. coli* [2]. Sequences were annotated with Prokka version 1.14.6 (https://github.com/tseemann/prokka). Coding sequences (CDS), represented with arrows, having identical length and 100% nt similarity are connected with black connections. Arrows with a given colour correspond to the same CDS. Considering the chromosomal locus of *S*. Rissen 2023–257642–001–01 as a reference, eight CDSs could be identified that were present in more than one member of the alignment. They corresponded to the following elements: IS6-like element IS26 family transposase, Tn3 family transposase ISEc63, Tn3 family transposase IS3000, OXA-48 family carbapenem-hydrolysing class D beta-lactamase OXA-181, CDS_1 replication initiation protein, ISKra4-like element ISKpn19 family transposase, DNA-invertase hin, CDS_3 hypothetical protein. All eight elements have identical length and 100% similarity across the whole alignment except Tn3 family transposase IS3000 which is present in three lengths. The longest (2,925 bp), represented in orange, is present in five members of the alignment (e.g. 2023–050284–001–01), while the green (2,859 bp), detected in two members (e.g. 2023–257642–001–01), and the violet (1,468 bp), present in six members (e.g. 2023–005381–004–01), are truncated forms of the longest at the N-terminal. The Tn3 family transposase ISEc63 is missing in six sequences.

Six *bla*_OXA-181_-positive isolates of MVST from pigs formed a clonal group (0–13 SNPs) with other isolates not carrying *bla*_OXA-181_, including eight from cattle and one from poultry (Figure 2B). The six pig isolates were from samples taken within 12 months from three different industrial pig farms belonging to the same agricultural company. Five *bla*_OXA-181_-positive isolates carried the same IncX1 plasmid harbouring *bla*_OXA-181_, while one isolate had a truncated form of the plasmid (Figure 3). These data suggest that this clone is endemic in livestock, and it recently acquired *bla*_OXA-181_ in the pig sector, where the resistance gene was able to persist at detectable level.

A clone of *S*. London included two isolates with the resistance gene from samples from the same pig farm and four isolates without the gene were from samples from food, an infected person and another pig farm (0–10 SNPs) (Figure 2C). We compared *bla*_OXA-181_-carrying plasmids from the two *S*. London isolates with resolved IncX1 plasmids (n = 15) from OXA-181-producing *E. coli* collected 2021–2023 within the EU-harmonised AMR monitoring programme [7]. The two *S*. London plasmids were almost identical, differing only by two SNPs and one single bp deletion, to IncX1 plasmid pMOL6975 (NCBI reference NZ_OX211907.1) [2]. This plasmid was detected in *E. coli* isolates from a different pig farm in the same area as the two *S.* London isolates [2], indicating its likely inter-species transfer.

Overall, *bla*_OXA-181_ was plasmid-borne in all the isolates except for *S*. Rissen, where it was chromosomal (Table, Figure 3). In three replicates, plasmid conjugation ability according to Møller et al. [12] was demonstrated in isolate 2023–0502284–001–01 showing a mean rate of 0.034 (standard deviation = 0.009) to the recipient laboratory strain of *E. coli* J53. Interestingly, aligning the chromosomal locus of *S*. Rissen hosting *bla*_OXA-181_ and the plasmids carrying this gene, we identified a set of genes with high across-isolates similarity, including *bla*_OXA-181_, shared by all the analysed plasmids and the *S*. Rissen chromosome. This set likely corresponds to the elementary unit involved in the spread of *bla*_OXA-181_ among the investigated genomes (Figure 3).

## Discussion

We detected 16 isolates carrying *bla_OXA-181_*-gene in four serovars. Ten isolates belonged to the multidrug-resistant (MDR) and pathogenic MVST, representing a possible emerging public health threat [13]. Although already established in commensal *E.* *coli* in pigs in Italy at least since 2021 [2], the emergence of carbapenemase-coding genes in SE represents a possible new direct health risk.

The *bla_OXA-181_* gene was first detected in animal isolates, then in human isolates, which strengthens our hypothesis that this pathogen was transmitted from animals or food to humans. Pigs might have become a reservoir for the resistance gene. The role of manure in agricultural practice should also be considered in the dissemination of this and similar resistance genes.

Involvement of different types of plasmids and even a chromosomal localisation are further indicators of the active dissemination. In pigs, we saw the emergence and persistence of MDR clones and the plasmid transmission between *E. coli* and SE, which likely occurred in the intestinal microbiota of Italian pigs. Moreover, the findings highlighted the critical importance of having human-animal integration and high-coverage in WGS surveillance, to timely identify emerging microbiological risks with reasonable sensitivity and to deeply investigate their epidemiology.

## Conclusion

*Salmonella enterica* with *bla*_OXA-181_ gene may have recently emerged in the human population of northern Italy, just a few months later than it was detected in the pig population (2023), as a likely consequence of zoonotic transmission along the food chain. The timing of these findings and their repeated occurrence suggest that this may represent an emerging public health issue for Italy and beyond, considering the role of the country as food producer and exporter. Furthermore, our findings have shown that continued One Health surveillance on large numbers of isolates is fundamental for the early identification of possible emerging threats, demonstrating once more its importance in public health.
