# Author’s correction for Euro Surveill. 2025;30(13)

**DOI:** 10.2807/1560-7917.ES.2025.30.14.250410c

**Published:** 2025-04-10

**Authors:** Eurosurveillance editorial team

**Affiliations:** 1 European Centre for Disease Prevention and Control (ECDC), Stockholm, Sweden

In the article entitled ‘Emergence of Salmonella enterica carrying blaOXA-181 carbapenemase gene, Italy, 2021 to 2024’ by Bolzoni et al., published on 3 April 2025, the second affiliation of Sara Angelone was missing at the time of publication. This mistake was corrected on 10 April 2025 at the request of the authors.

